# From Worms to Drug Candidate: The Story of Odilorhabdins, a New Class of Antimicrobial Agents

**DOI:** 10.3389/fmicb.2019.02893

**Published:** 2019-12-18

**Authors:** Emilie Racine, Maxime Gualtieri

**Affiliations:** Nosopharm, Nîmes, France

**Keywords:** Odilorhabdins, antimicrobial agent, *Xenorhabdus*, translation inhibitor, cationic peptide

## Abstract

A major issue currently facing medicine is antibiotic resistance. No new class of antibiotics for the treatment of Gram-negative infections has been introduced in more than 40 years. We screened a collection of *Xenorhabdus* and *Photorhabdus* strains in the quest to discover new structures that are active against the most problematic multidrug-resistant bacteria. These species are symbiotic bacteria of entomopathogenic nematodes and their life cycle, the richness of the bacteria’s genome in non-ribosomal peptide synthetase (NRPS) and polyketide synthase (PKS) genes, and their propensity to produce secondary metabolites with a large diversity of chemical structures make them a good starting point to begin an ambitious drug discovery program. Odilorhabdins (ODLs), a novel antibacterial class, were identified from this campaign. These compounds inhibit bacterial translation by binding to the small ribosomal subunit at a site not exploited by current antibiotics. Following the development of the total synthesis of this family of peptides, a medicinal chemistry program was started to optimize their pharmacological properties. NOSO-502, the first ODL preclinical candidate was selected. This compound is currently under preclinical development for the treatment of multidrug-resistant Gram-negative infections in hospitalized patients.

## Introduction

The efforts of the pharmaceutical industry to generate new highly potent antibiotics with novel mechanisms of action have weakened dramatically over the last three decades for economic, scientific, or strategic reasons, resulting in the decline of the discovery of new classes of antibacterials. The rapid emergence of resistant bacteria combined with the absence of new drugs has led clinicians to a therapeutic impasse, especially in intensive care units. It is thus urgent to find new antibiotic chemical scaffolds to renew the current therapeutic arsenal. Natural products have historically been of crucial importance in the identification and development of antibacterial agents (Clardy et al., 2006). Indeed, most antibiotics in clinical use or advanced development come from secondary metabolites that were originally isolated from bacteria or fungi, such as penicillin, isolated from the fungus *Penicillium*, or tetracycline found in the soil-dwelling bacteria *Streptomyces aureofaciens*. Historically, *Actinomycetes* have been the most important source for the discovery of new antibiotics. Many drugs used today in clinical practice originate from metabolites produced by *Actinomycetes*, (Genilloud, 2017) such as chloramphenicol (producing strain: *Streptomyces venezuelae*), daptomycin (*Streptomyces roseoporus*), erythromycin (*Saccharopolyspora erythraea*), gentamicin (*Micromonospora purpurea*), lincomycin (*Streptomyces lincolnensis*), rifamycin (*Amycolatopsis mediterranei*), streptomycin (*Streptomyces griseus*), tetracycline (*S. aureofaciens*), and vancomycin (*Amycolatopsis orientalis*). Although *Actinomycetes* are still one of the most important sources for novel structures, (Genilloud, 2017) it is equally important to explore new underexploited microbial bioresources, as shown by the recent characterization of two highly promising antibiotics with novel mechanisms of action. Teixobactin was isolated from uncultured *Eleftheria terrae*, (Ling et al., 2015) and Odilorhabdins (ODLs) from *Xenorhabdus nematophila* (Pantel et al., 2018).

## *Xenorhabdus* and *Photorhabdus*: Important Strains for the Discovery of New Antibacterials

**FIGURE 1:**
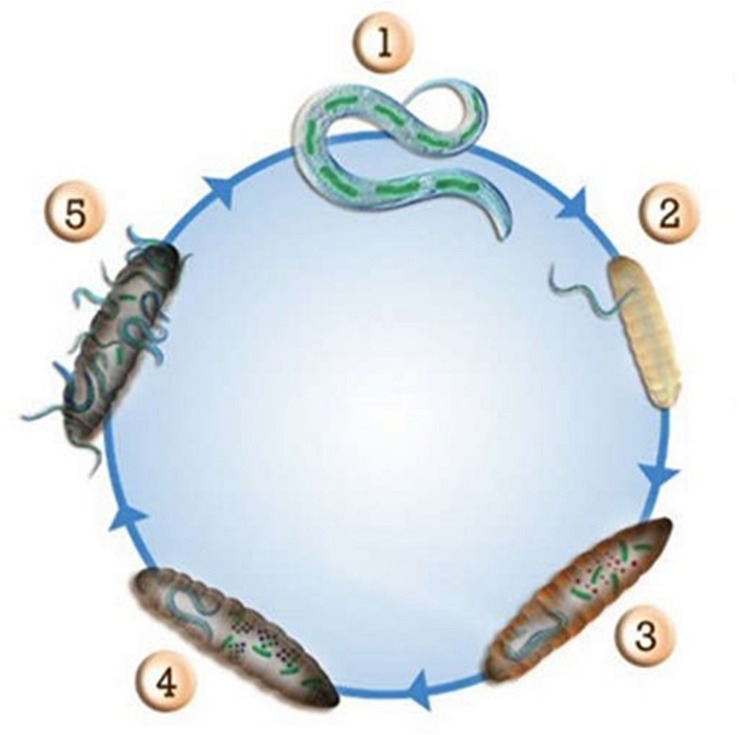
Life cycle of entomopathogenic nematodes and their symbiotic bacteria in insect larvae. (1) *Photorhabdus* or *Xenorhabdus* bacteria live in the intestine of the host nematode; (2) The host nematode infects an insect; (3) *Photorhabdus* or *Xenorhabdus* bacteria are released within the insect and produce compounds to kill it; (4) *Photorhabdus* or *Xenorhabdus* bacteria produce a set of antibiotic molecules to prevent microbial competitors of the environment to degrade the corpse of the insect; (5) The nematode and the bacteria use the biomass of the insect as nutrients to reproduce. *Photorhabdus* or *Xenorhabdus* bacteria recolonize the nematode that will emerge from the corpse of the insect.

The genera *Xenorhabdus* and *Photorhabdus* of the *Enterobacteriaceae* family are mutualistically associated with entomopathogenic nematodes. These bacteria have a fascinating life cycle (Figure 1) that requires the production of a great diversity of antibacterial and antifungal compounds (Dreyer et al., 2018). The bacteria-nematode pair infects and kills insects. *Xenorhabdus* or *Photorhabdus* bacteria then establish suitable conditions for the reproduction of the nematode by providing nutrients and protecting the insect corpse from environmental predators, such as bacteria and fungi. *Xenorhabdus* and *Photorhabdus* offer many advantages for anti-infective drug discovery. First, they produce novel and undescribed antimicrobial molecules with original chemical structures. This feature is genetically supported by the high content of non-ribosomal peptide synthetase (NRPS) and polyketide synthase (PKS) genes in their genomes (Tobias et al., 2017). NRPS and PKS enzymes or hybrids thereof are responsible for the biosynthesis of complex secondary metabolites via the combinatorial assembly of simple blocks, such as amino acids, acetate, or propionate. Then, antimicrobial compounds produced by *Xenorhabdus* and *Photorhabdus* interact with the biological matrices of the dead insect but are not toxic for the nematode. These properties represent a natural primary filter for compound drugability and safety in eukaryotic organisms. Finally, these genera are an underestimated and neglected source of novel bioactive compounds and thus constitute a promising source for undisclosed and unpatented molecules.

## Discovery of Odilorhabdins

Most bacteria do not express their full genomic potential under laboratory growth conditions or when using classic culture media and some secondary metabolites may not be produced. Multiple strategies to increase metabolite production have been described. A simple approach consists of culturing bacterial strains in various media and altering various cultivation parameters, such as temperature, salinity, flask shape, and aeration. A second approach is based on a published study showing that mutations in the RNA polymerase of various *Actinomycetes* strains are effective in enhancing the production of new antibiotics by activating biosynthetic gene clusters, which are “silent” or poorly expressed in wild type strains (Tanaka et al., 2013). This strategy has been applied to *Xenorhabdus* and *Photorhabdus* strains by the cultivation of selected clones resistant to rifamycins, an antibiotic family known to generate characteristic mutations on the RNA polymerase β subunit. Finally, a third approach, based on cocultivation of the strain of interest with multiple microbial strains (e.g., *Streptomyces abikoensis*, *Streptomyces glomeroaurantiacus*, *Alteromonas macleodii*, *Micromonospora aurantiaca*, *Stenotrophomonas terrae*, *Bacillus subtilis*, *Staphylococcus aureus*, and *Sphingomonas aquatilis*) has been used for antibiotic production and is well documented in the literature (Ueda and Beppu, 2017). For example, biphenomycin C was produced by *Streptomyces griseorubiginosus* and converted to the active antibiotic in a coculture with *Pseudomonas maltophilia* (Ezaki et al., 1993). In another example, alchivemycin A was only produced when *Streptomyces lividans* was in direct contact with bacteria producing mycolic acid (Onaka et al., 2011).

These three strategies were applied to *Xenorhabdus* and *Photorhabdus* bacteria and the supernatants of 150 cultured strains were screened for the presence of antibacterial activity. Active supernatants were fractionated by high-performance liquid chromatography (HPLC). Antibacterial, antifungal, and cytotoxic activities of the obtained fractions were measured to select those showing only antibacterial activity to avoid potentially cytotoxic compounds. The isolation and identification of ODLs from one of these fractions followed a traditional protocol, including isolation/purification by HPLC and molecular mass determination by mass spectrometry. A total of three antibacterial metabolites were characterized from the culture supernatant of *X. nematophila* strain CNCM I-4530. These compounds were named NOSO-95A (MW: 1,296 Da), NOSO-95B (MW: 1,280 Da), and NOSO-95C (MW: 1,264 Da) (Pantel et al., 2018). The molecular masses of ODLs were compared with those in the antibiotic mass bank to confirm their novelty. ODLs have a wide antibacterial activity spectrum that includes Gram-negative and Gram-positive bacteria. NOSO-95A was shown to have activity against many resistant strains with limited treatment options, such as carbapenem-resistant *Enterobacteriaceae* (CRE) or methicillin-resistant *Staphylococcus aureus* (MRSA) (Gualtieri et al., 2013; Pantel et al., 2018). At the same time, NOSO-95A did not show any cytotoxic effect on human HepG2 cell line, even at concentrations up to 128 μg/mL which exceeds the typical MICs for *Escherichia coli*, *Klebsiella pneumoniae*, and *S. aureus* by 16-, 32-, and 128-fold, respectively. NOSO-95A showed bactericidal activity against *K. pneumoniae* and *S. aureus*. These encouraging results prompted us to continue the characterization of NOSO-95A. Its *in vivo* efficacy was evaluated in a mouse septicemia model with *S. aureus*. All NOSO-95A-treated mice survived up to the end of the study (120 h) at a dose of 2.5 mg/kg. These data show the high potential of ODLs as a new class of antibiotic.

## Structural Determination and Identification of the Biosynthetic Gene Cluster

**FIGURE 2:**
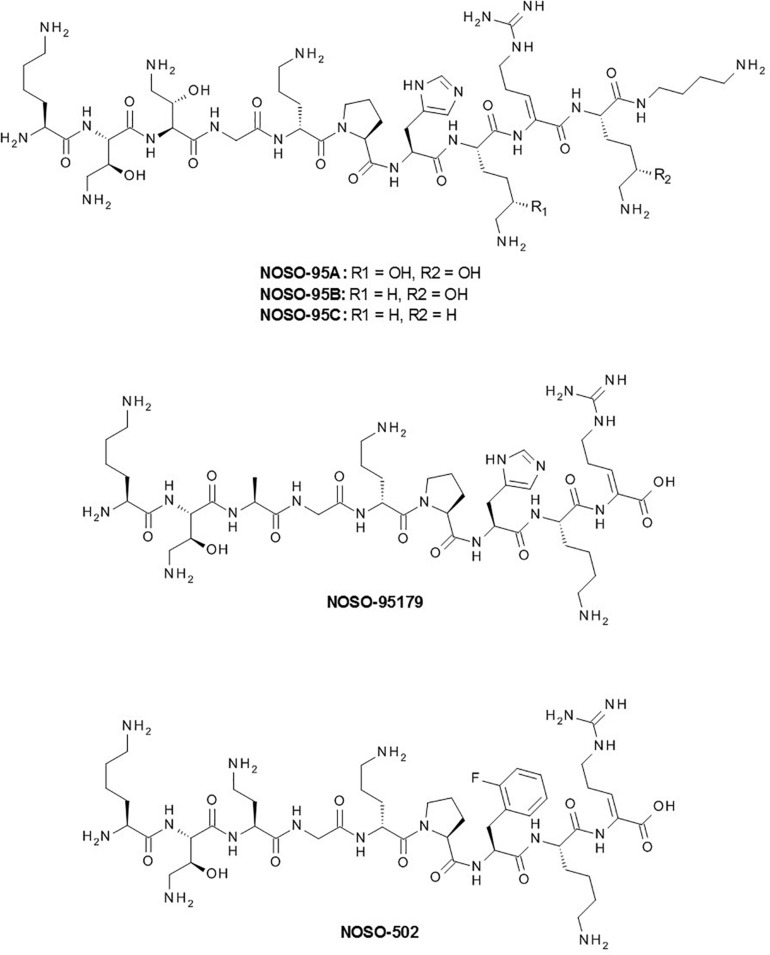
Chemical structures of NOSO-95A, B, and C, NOSO-95179 and NOSO-502.

The chemical structure of NOSO-95A, solved by nuclear magnetic resonance (NMR) and LC-MS/MS fragmentation analysis, revealed ODLs to be representative of a new chemical class of antibiotics (Figure 2) (Pantel et al., 2018). NOSO-95A is a 10-mer linear cationic peptide containing four proteinogenic and six non-standard amino acids: (2S,3S)-α,γ-diamino β-hydroxybutyric acid (Dab(βOH) at positions 2 and 3, D-ornithine (D-Orn) at position 5, Z-α,β–dehydroarginine (Dha) at position 9, (5S)-5-hydroxylysine (Dhl) at positions 8 and 10, and a functionalized secondary amide at the C-terminal position [α,δ–diamino butane (Dbt)]. NOSO-95B and C differ from NOSO-95A by the substitution of Dhl at position 10 (NOSO-95B) or at positions 8 and 10 (NOSO-95C) by a lysine (Figure 2). We developed the synthesis of all diastereoisomers of the three non-standard amino acids, Dab(βOH), Dha, and Dhl, and elucidated the stereochemistry of each chiral center of NOSO-95A by the advanced Marfey’s method (Bhushan and Brückner, 2004). Orn was found to be the only amino acid of R configuration while all other chiral centers were of S configuration. At the same time that the current study was being conducted, we identified the biosynthetic NRPS gene cluster (Pantel et al., 2018). This step was necessary for the production of ODLs and related analogs by engineering NRPS module enzymes. This strategy was used for daptomycin, a cyclic lipopeptide antibiotic used for the treatment of infections caused by *S. aureus* (Nguyen et al., 2010). We identified four large NRPS-coding genes in the genome of the producer *X. nematophila* as the putative biosynthetic gene cluster using anti-SMASH, a secondary metabolite gene cluster prediction software. Inactivation of the first gene of the cluster abolished production of all three ODLs, confirming that the cluster is responsible for their production (Pantel et al., 2018). Two of the non-standard amino acids, Dab(βOH) and Dha, were not commercially available and were required for the total synthesis of NOSO-95C. We developed their synthesis on a multi-gram scale and reported the first total synthesis of NOSO-95C (Sarciaux et al., 2018). The ^1^H NMR and LC-MS spectra and antibacterial activity of the natural and synthetic peptides were similar.

## Structure-Activity Relationships and Selection of a Preclinical Candidate

**FIGURE 3:**
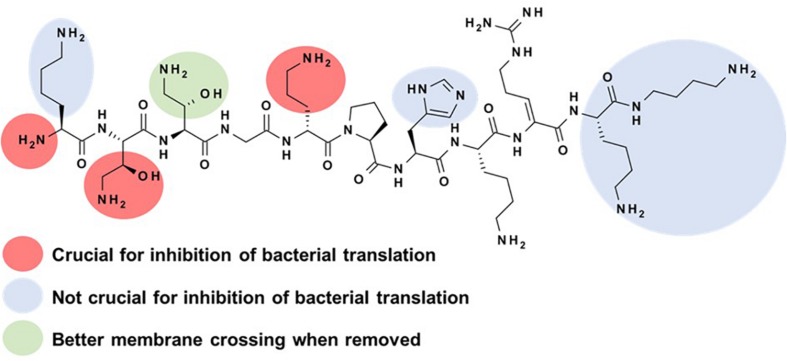
Structure-activity relationships for the inhibition of bacterial translation by NOSO-95C.

Based on these encouraging results and with a validated synthetic method, we initiated a medicinal chemistry program on NOSO-95C to study the structure-activity relationships (SAR) of ODLs with the objective to better understand the role of each amino acid for the antibacterial activity and the inhibition of bacterial translation (Sarciaux et al., 2018). Before starting this study, the inhibition of bacterial translation was identified as the principal mode of action of ODLs. We evaluated the antibacterial activity of analogs against *Enterobacteriaceae* (*E. coli* and *K. pneumoniae*) and their potential to inhibit bacterial translation to help drive preliminary SAR studies. First, alanine scanning (Ala scan) was performed. This consists of replacing each amino acid by an alanine to evaluate the impact of lateral chains on biological activity. This strategy has been previously used to decipher the SAR of various antibacterial peptides, such as an analog of teixobactin, (Parmar et al., 2017) and feglymycin (Hänchen et al., 2013). Removal of the lateral chain of Lys1, His7, Lys8, Lys10, or Dbt11 had little or no impact on the antibacterial activity of NOSO-95C and inhibition of translation. Replacing Dab(βOH)3 by alanine resulted in a four-fold gain in antibacterial activity and in the same level of inhibition of bacterial translation than that of NOSO-95C. A better passage through the bacterial membrane could explain the improvement of antibacterial activity while conserving the same level of inhibition of translation (Figure 3). Significant decreases of both antibacterial activity and inhibition of the translation were observed when substituting Dab(βOH)2 and D-Orn5 (Figure 3). As shown later on using X-ray crystallography, the lateral chains of these two amino acids interact directly with the bacterial ribosome (Pantel et al., 2018). Substitution of Gly4, Pro6, and Dha9 by an alanine should have a strong effect on the secondary structure of the peptide. Indeed, the antibacterial activity of these analogs and the inhibition of translation were strongly reduced with the exception of the analog in which Pro6 was replaced by Ala, for which activity was conserved on *E. coli* while measuring a five-fold decrease of the inhibition of translation. This could be explained by a more flexible structure, making it easier for this analog to cross the bacterial membranes. We next investigated the effect of modifying the lateral chain of Dab(βOH)2 and Dha9. First, the hydroxyl group of Dab(βOH)2 was removed resulting in a strong decrease of both antibacterial activity and inhibition of translation. The same deleterious effect was observed when Dab(βOH)2 was replaced by allo-threonine (AlloThr) or Ser to study the influence of the amine function of the lateral chain. Influence of substitution of the lateral chain of Dha9 and removal of the double bond was next investigated by introducing dehydroamino butyric acid (DhAbu), L- or D-Arg. These substitutions had a limited impact, unlike substitution by alanine, meaning that either the double bond or the guanidine moiety is needed for efficient antibacterial activity and inhibition of translation. Finally, truncation of amino acids from the N- and C-terminal position of the peptide was tested to find the shortest active sequence. Removing amino acids from C-terminal part up to Lys10 (included) led to structures with almost identical *in vitro* antibacterial efficacy and slightly better inhibition of bacterial translation compare to parent compound (Figure 3). Importance of Dha9 was confirmed by the strong decrease of antibacterial activity and inhibition of the translation of the analog in which this amino acid was removed. Reduction of the peptidic chain from the N-terminal side was then investigated. Removal of the first amino acid or both Lys1 and Dab(βOH)2 was detrimental for both antibacterial efficacy and inhibition of translation. The key role of the N-terminal amine function for the binding to the ribosome was later confirmed by co-crystallization studies.

The combination of best modifications and of the shorter active sequence led to NOSO-95179 identified as a lead compound showing promising *in vitro* and *in vivo* antibacterial activity against *Enterobacteriaceae* (including CRE) and low toxicity (Pantel et al., 2018; Sarciaux et al., 2018). This compound was used as the starting point for a lead-optimization campaign. The object of this final drug discovery phase was to maintain the favorable properties of the lead compound while improving on any deficiencies. One important objective was to enhance *in vitro* antibacterial activity and *in vivo* efficacy. Five hundred analogs were synthesized and evaluated using assays similar to those performed during the previous stage (MIC determination against a panel of referenced strains, cytotoxicity against the HepG2 cell line, and inhibition of bacterial translation). None of the compounds displayed cytotoxic activity and the 10% with the best antibacterial activity and inhibition of bacterial translation were selected and examined for extended microbiological properties (MIC against a large panel of bacterial strains displaying different resistance profiles, bactericidal kinetics, and frequency of resistance), and ADME-tox properties (blood stability, acute toxicity in the mouse, and hemolytic properties). The pharmacokinetic (PK) properties, plasma protein binding, and efficacy in a murine sepsis infection model of 40 selected analogs were then assessed. Finally, the properties of the 10 best analogs were compared using a large panel of tests, including pharmacology, ADME, and safety toxicology studies. Analogs with modifications at both the Ala3 and His7 positions showed a combination of good *in vitro* activity and improved *in vivo* efficacy through favorable PK profiles, leading to increased exposure. Among these analogs, NOSO-502 showed the best profile and was selected as a promising candidate (Figure 2) (Racine et al., 2018). MIC of NOSO-502 ranges from 0.5 to 4 μg/ml against *Enterobacteriaceae* type strains and CRE strains expressing carbapenemase belonging to classes A, B, and D of the Ambler classification. This compound retains excellent activity against strains resistant to colistin, a last resort antibiotic used to treat multidrug-resistant bacterial infections, through expression of mobile colistin resistance (mcr) genes or mutations in mgrB, PmrAB, PhoPQ genes. In addition, NOSO-502 has a low potential for resistance development (Racine et al., 2018). It is effective in mouse models of serious hospital-acquired infections (sepsis, complicated urinary tract infection (UTI), lung infection) and provides significant protection against *E. coli* and *K. pneumoniae*, the highest-incidence hospital pathogens in complicated intra-abdominal infection (IAI) and UTI. Interestingly, NOSO-502 was effective against an *E. coli* strain that expresses the metallo-β-lactamase NDM-1 and is resistant to other major antibiotic classes. Although nephrotoxicity and cardiotoxicity are associated with many antibiotics, NOSO-502 exhibited a good safety profile based on data from *in vitro* nephrotoxicity, cardiotoxicity, genotoxicity, or cytotoxicity standard studies. AUC/MIC was selected as the PK/PD index that shows the highest correlation with the antibacterial effect of NOSO-502 in a murine thigh infection model (Zhao et al., 2018). These data combined with human PK exposure and MIC distribution will be helpful in determining an appropriate dosing-regimen for future clinical studies.

## Mechanism of Action

Deciphering the mechanism of action of a new antibacterial compound is crucial for a drug discovery and development program. However, it is often a complicated process, for which various strategies have been described (Farha and Brown, 2016). We initially investigated the mode of action of ODLs by assessing the effect of the compound on the incorporation of radiolabeled precursors into four major biosynthetic pathways (protein, RNA, DNA, or peptidoglycan synthesis) of *E. coli* cells. These experiments demonstrated that bacterial protein synthesis is the main target of ODLs. In accordance with this conclusion, we showed that ODLs inhibit the production of a protein in an *E. coli* cell-free transcription-translation system with an IC_50_ in the same range as that of known ribosome-targeting antibiotics, such as chloramphenicol and spectinomycin (Pantel et al., 2018). Target identification was conducted by selecting resistant mutants carrying alterations in the drug-target site. As ODLs interfere with protein synthesis, one of the putative targets was the ribosome, which is the major target for antibacterials. The ribosome is a supramolecular enzyme which translates genetic information into proteins. In bacteria, it is composed of two subunits, a small 30S and large 50S subunit, which join to form a 70S ribosome. Each subunit is composed of proteins and ribosomal RNA (rRNA). In wild type *E. coli*, seven copies of the rRNA operon are present in the chromosome. An *E. coli* strain in which six of the seven rRNA operons had been deleted was used to increase the chances of selecting resistant strains with mutations in the rRNA. Whole-genome sequencing of the selected ODL-resistant clones showed that almost all the mutants carried mutations in the 16S rRNA gene of the small ribosomal subunit, clustered in the vicinity of the decoding center (DC) (Pantel et al., 2018). The DC of the ribosome performs messenger RNA (mRNA) translation and provides the fidelity of the codon/anticodon interactions, along with performing mRNA translocation during protein biosynthesis.

**FIGURE 4:**
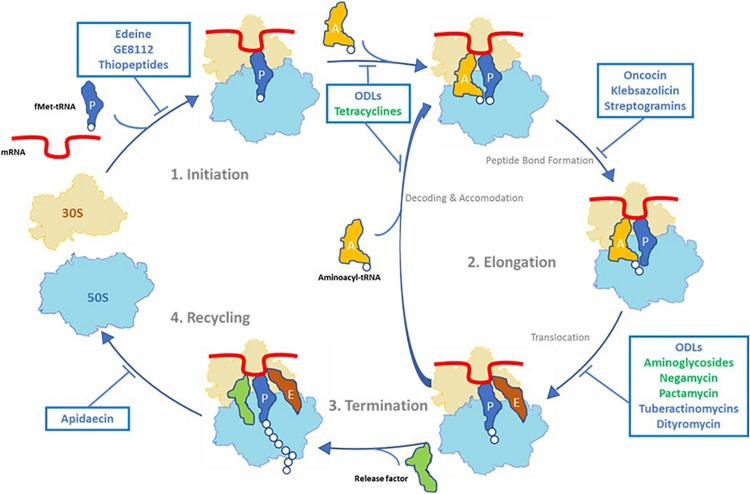
Overview of antibiotics that target the 30S ribosomal subunit (in green) and antibacterial peptides that inhibit the prokaryotic translation cycle (in blue).

The crystal structure of a 70S ribosome associated with ODLs confirmed that this new class of antibiotic interacts with the small ribosomal subunit (30S) by forming multiple hydrogen bonds with 16S rRNA residues of helices 31, 32, and 34 (h31, h32, and h34) (Pantel et al., 2018). This binding site is at a site distinct from those of other inhibitors that target the 30S ribosomal subunit, such as aminoglycosides, tuberactinomycins (viomycin, capreomycin), edeine, pactamycin, tetracycline, and negamycin (Table 1). The tetracycline and negamycin sites are closest to the site of ODL binding but do not overlap with that of ODLs. Moreover, ODL-resistant clones are not resistant to other clinically used ribosomal inhibitors. These results were crucial for carrying the development of ODLs forward. In this binding site, ODLs simultaneously interact with the 16S rRNA and the anticodon loop of the transfer RNA (tRNA) in the A-site, suggesting that the drug may increase the affinity of the aminoacyl tRNA for the ribosome. The predictable consequence is constrained progression of the ribosome along the mRNA and decreased fidelity of translation. We analyzed the effect of ODLs on translocation using a toe-printing assay. This method allows the determination of interactions between ribosomes or ribosomal subunits and mRNA. At lower concentrations, ODLs induced amino acid misincorporation by reducing the accuracy of decoding, whereas at higher concentrations they interfered with translocation. This mode of translation inhibition that is dependent on the drug concentration is similar to that described for aminoglycosides and negamycin. However, these antibiotics achieve this effect via different mechanisms. Aminoglycosides interact exclusively with the 16S rRNA and increase tRNA affinity by stabilizing a conformation of the 16S rRNA that interacts with the tRNA anticodon (Demirci et al., 2013). In contrast, negamycin and ODLs establish direct contacts with the A-site tRNA, but they interact at different sites (Pantel et al., 2018). Antimicrobial peptides (AMPs) that target the ribosome are rare. Indeed, most AMPs are mostly known for their disruptive effects on bacterial membranes (e.g., polymyxin B, gramicidin, LL-37, and melittin). Only nine classes interact with the bacterial ribosome, of which five, including ODLs, interact with the small ribosomal subunit (Polikanov et al., 2018). The binding site on the 30S ribosomal and mode of action of ODLs are different from the four other classes (Figure 4). Edeine (EDE), a pentapeptide amide produced by *Bacillus brevis*, and GE81112, a tetrapeptide produced by some *Streptomyces* species, target the translation initiation phase. EDE inhibits the formation of the 30S pre-initiation complex and prevents the formation of a competent 70S initiation complex, whereas GE81112 induces conformational changes of the 30S subunit, preventing its joining with the 50S subunit to form the 70S initiation complex (Dinos et al., 2004; Brandi et al., 2006). Similarly to ODLs, dityromycin (DIT) and tuberactinomycins (TUBs) inhibit translocation at the elongation phase, but by different mechanisms. DIT, a cyclic peptide produced by *Streptosporangium cinnabarum*, interacts with the 30S ribosomal protein S12 and inhibits translocation by preventing EF-G from adopting the final state necessary for translocation of the tRNAs and mRNA on the small subunit (Bulkley et al., 2014). TUBs, cyclic pentapeptides produced by various *Streptomyces* species, bind at the interface between the 30S and 50S subunits and act by trapping the ribosome in an intermediate state on the translocation pathway (Ly et al., 2010). The targets of the four classes of AMPs that target the 50S ribosomal subunit are mostly clustered around the peptidyl transferase center (PTC), where the peptide bond is formed. Streptogramins, a class produced by *Streptomyces* species, prevent correct positioning of tRNAs into the PTC, making peptide-bond formation impossible (Schmeing et al., 2005). Klebsazolicin, produced by *K. pneumoniae*, obstructs the ribosomal exit tunnel and blocks elongation of the nascent peptide (Metelev et al., 2017). Proline-rich antimicrobial peptides (PrAMPs) have been found in insects, crustaceans, and mammals. Oncocins, the best-characterized type I PrAMPs, bind within the ribosomal exit tunnel in a reverse orientation relative to the nascent polypeptide chain, preventing transition into the elongation phase of translation (Roy et al., 2015). The apidaecin derivative Api137 blocks translation termination by trapping release factors (Florin et al., 2017). Finally, thiopeptide antibiotics GE2270A and thiostrepton inhibit translation by interacting with the translation factor EF-Tu or directly with the ribosome. Thiopeptides binding site on the large ribosomal subunit overlaps the IF2, EF-Tu, and EF-G translation factors’ binding site (Bagley et al., 2005).

**TABLE 1:** Binding site on the 30S ribosomal subunit of various antibiotics.

**Antibiotic**	**Binding site on the 30S and 50S ribosomal subunit**	**References**
ODLs	h31, h32, h34	Pantel et al., 2018
Aminoglycosides	h44	Wilson, 2009
Neomycin	h44, H69	Wang et al., 2012
Tuberactinomycins	h44 and H69	Stanley et al., 2010
Edeine	h24, h28, h44, h45	Pioletti et al., 2001
Pactamycin	h23, h24	Brodersen et al., 2000
Tetracycline	h31, h34	Brodersen et al., 2000
Negamycin	h34	Polikanov et al., 2014

*h: helix of the 30S subunit; H: helix of the 50S subunit.*

## Antibiotics That Are Active Against Carbapenem-Resistant *Enterobacteriaceae* (CRE) in Development or Recently Approved

In 2013, CRE, *Clostridium difficile*, and *Neisseria gonorrhoeae* bacteria were identified by the Centers for Disease Control and Prevention (CDC) as urgent drug-resistant threats, confirming the need to rapidly develop new antibiotic scaffolds active against these pathogens (Centers for Disease Control and Prevention, 2013). The urgent need to find new therapies against CRE was recently confirmed by the WHO (Tacconelli et al., 2018). Carbapenems are broad-spectrum β-lactam antibiotics considered to be the last treatment option for infections caused by multidrug-resistant pathogens. However, their use has been compromised due to the increasing prevalence of CRE infections in hospitals. CRE have become increasingly resistant to last-resort antibiotics and cause a range of healthcare-associated infection, such as pneumonia (HABP/VABP), UTI, IAI, and bloodstream infection (BSI) (Sheu et al., 2019). Numerous outbreaks of hospital acquired infections due to CRE were reported in different regions of the world (Tzouvelekis et al., 2012; van Duin and Doi, 2017). Recent reports show that the mortality of patients with CRE infections is three times higher than for patients with similar susceptible infections (Martin et al., 2018). In 2013, the CDC evaluated that CRE (*E. coli* and *K. pneumoniae*) were responsible of 9,300 nosocomial infections in the United States and caused the deaths of approximately 600 people (Centers for Disease Control and Prevention, 2013). The main mechanism responsible for carbapenem resistance among *Enterobacteriaceae* is the production of a carbapenemases, which are β-lactamase enzymes that efficiently break down carbapenem antibiotics. Many carbapenemases belonging to Ambler classes A, B, and D of β-lactamases have been described in *Enterobacteriaceae*. Optimal treatment for serious infections due to CRE is yet to be determined, but current treatment often involves the use of gentamicin, tigecycline, colistin, and amikacin, alone or in combination with each other antibiotics and carbapenems (Martin et al., 2018). In such infections, resistance to carbapenems is associated with high levels of resistance to other antibiotics [e.g., colistin (22.6%), gentamicin (43.5%), tigecycline (15.2%)] (Trecarichi and Tumbarello, 2017). Furthermore, certain therapies, such as colistin, gentamicin, or amikacin, are associated with an increased risk of nephrotoxicity. These observations highlight the importance of the development of new therapeutic options to obtain more positive outcomes in CRE infections, especially compounds from new classes of antibiotic with original mode of action to limit the risk of cross-resistance.

NOSO-502 or other ODL analogs could be one such novel therapy. Indeed, this new family of antibiotics is active against CRE isolates producing β-lactamases belonging to Ambler classes A, B, C, and D and also exhibiting resistance to gentamicin, amikacin, polymyxin B, or tigecycline. The vast majority of agents recently approved or still in development are the result of modifications of existing agents (Table 2). They cannot generally overcome multiple existing resistance mechanisms. Furthermore, none of the recently approved antimicrobials are effective against all classes of carbapenemases, unlike ODLs, with the exception of eravacycline, (Livermore et al., 2016) an antibiotic of the tetracycline class approved by the United States Food and Drug Administration (FDA) in 2018 for the treatment of complicated IAI. The combination of ceftazidime + avibactam (Avycaz^®^) was approved by the FDA in 2015 and by the European Medicines Agency in 2016 for the treatment of cUTI and cIAI. It is active against isolates producing class A and D carbapenemases but does not cover metallo-β-lactamases like NDM-1, VIM, or IMP (Falcone and Paterson, 2016). The combination of meropenem + vaborbactam (Vabomere^®^), approved by the FDA in 2017 for the treatment of cUTI (including acute pyelonephritis), cIAI, and HABP/VABP, is only active against class A carbapenemase-producing strains (KPC) within the CRE group (Castanheira et al., 2017). Plazomicin is an aminoglycoside that was approved in June 2018 for the treatment of cUTI (including pyelonephritis) caused by *E. coli*, *K. pneumoniae*, *Enterobacter cloacae*, and *Proteus mirabilis*. As other aminoglycosides, its activity is not affected by resistance mechanisms to other antibiotic classes, such as β-lactamases and carbapenemases, including metallo-β-lactamases. However, it has been shown that the class B metallo-β-lactamase NDM-1 is frequently co-expressed with 16S ribosomal RNA methyltransferases and plazomicin, like all aminoglycosides, is inactive against strains that express these enzymes (Serio et al., 2019). Other drug candidates to address these resistance issues are currently under clinical development but all belong to existing classes of antibiotics (β-lactams, β-lactamase inhibitors combined with β-lactams, tetracyclines, aminoglycosides, fosfomycins and polymyxins). Nevertheless cefiderocol, a siderophore cephalosporin, (Kohira et al., 2015) and the combinations aztreonam-avibactam or cefepim-zidebactam appear to be promising options to treat such infections (Sader et al., 2017a, b).

**TABLE 2:** Antibiotics active against carbapenem-resistant *Enterobacteriaceae* (CRE) in development or recently approved by the FDA.

**Product**	**Company**	**Antibacterial class**	**Status**
Ceftazidime + avibactam	Allergan/Pfizer	βL	Approved by FDA in 2015
Vabomere	Melinta	βL-βLI	Approved by FDA in 2017
Plazomicin	Achaogen	Aminoglycoside	Approved by FDA in 2018
Eravacycline	Tetraphase	Tetracycline	Approved by FDA in 2018
Imipenem + cilastatin + relebactam	Merck & Co.	βL-βLI	Phase 3
Aztreonam + avibactam	Pfizer	βL-βLI	Phase 3
ZTI-01	Nabriva	Fosfomycin	Phase 3
Cefiderocol	Shionogi	Siderophore-βL	Phase 3
LYS228	Boston pharmaceuticals	Monobactam	Phase 2
AIC499	AiCuris	βL	Phase 1
Cefepime + Zidebactam	Wockhardt Ltd.	βL-βLI	Phase 1
GSK3342830	GlaxoSmithKline	Siderophore-βL	Phase 1
SPR741	Spero	Polymyxin	Phase 1
TP-6076	Tetraphase	Tetracycline	Phase 1

*βL: beta-lactam, βLI: beta-lactamase inhibitors.*

## Conclusion

The need for new antibiotic classes is exceedingly urgent. In this context, ODLs are a promising option to treat some of the most problematic multidrug-resistant Gram-negative infections. This is one more example of the importance of preserving biodiversity as a source of future drugs.

## Author Contributions

Both authors listed have made a substantial, direct and intellectual contribution to the work, and approved it for publication.

## Conflict of Interest

MG is a founder and shareholder of Nosopharm. ER and MG are employees of Nosopharm.
